# LB2304. INHALE WP3: Results of a multi-centre randomised controlled trial (INHALE) testing the utility of rapid multiplex PCR at point-of-care for the antibiotic management of hospital-acquired and ventilator-associated pneumonia in critical care.

**DOI:** 10.1093/ofid/ofac492.1894

**Published:** 2022-12-15

**Authors:** Virve Enne, Susan Stirling, Julie Barber, Juliet High, Charlotte Russell, Kerry Dresser, Zaneeta Dhesi, David Brealey, Suveer Singh, Ann Marie Swart, David Livermore, Vanya Gant

**Affiliations:** University College London, London, England, United Kingdom; University of East Anglia, Norwich, England, United Kingdom; University College London, London, England, United Kingdom; Norwich Clinical Trials Unit, Norwich, England, United Kingdom; University of East Anglia, Norwich, England, United Kingdom; University of East Anglia, Norwich, England, United Kingdom; University College London, London, England, United Kingdom; University College London Hospital, London, England, United Kingdom; Chelsea and Westminster Hospital, London, England, United Kingdom; University of East Anglia, Norwich, England, United Kingdom; University of East Anglia, Norwich, England, United Kingdom; University College London Hospital, London, England, United Kingdom

## Abstract

**Background:**

The FilmArray Pneumonia Panel (bioMérieux) is a rapid multiplex PCR aiming to increase speed and sensitivity in the microbiological diagnosis of pneumonia. This offers potential for improved antimicrobial stewardship and evidence-based antibiotic prescribing. Whether such gains can be realised in practise is uncertain, thus, we conducted a randomized controlled trial to examine this.

**Methods:**

Adults and children in ICU with suspected hospital-acquired or ventilator-associated pneumonia were randomized to standard care or a FilmArray Pneumonia Panel PCR test at the point of care supported by an optional prescribing algorithm. Participants were followed for 28 days. FilmArray tests were run retrospectively in the control-arm but results were not provided to treating clinicians. Antimicrobial therapy was adjudged by a committee of blinded, independent microbiologists for ‘activity’ and ‘proportionality’ vs. pathogens detected in each arm regardless of the time and method by which they were found. Clinical cure was not blinded and judged by site PI according to defined clinical parameters.

**Results:**

Between July 2019 and August 2021, 556 participants were randomized at 13 hospitals; 545 met eligibility criteria; 92 were children and 183 had COVID-19 at enrolment. Baseline characteristics were well balanced between study arms. In the intervention arm 76.5% of participants were judged to have received active and proportionate antibiotics at 24h vs. 55.9% in the control arm (p< 0.001); proportions at 72h were 73.4% and 58.8%, respectively p< 0.001). Site-reported clinical cure of pneumonia at 14 days was 56.7% in the intervention arm and 64.7% in the control arm (95% CI -0.15 to 0.02), with exploratory analyses suggesting site heterogeneity. 28-day mortality was 31.3% in the intervention arm and 28.2% in the control arm (p = 0.098). Median ventilator-free days by Day 21 were 2 days in both study arms. Median ICU length of stay was 14 days in both arms.

**Conclusion:**

Use of the FilmArray Pneumonia Panel PCR led to a significant increase in the proportion of patients receiving ‘active and proportionate’ antibiotic therapy at 24h. Reasons for lower reported clinical cure, despite better tailored antibiotic use, remain under active analysis.

**Disclosures:**

**Virve Enne, BSc PhD**, Biomerieux: Advisor/Consultant|Biomerieux: Advisor/Consultant|Biomerieux: Grant/Research Support|Biomerieux: Grant/Research Support|Inflammatix: Grant/Research Support **Juliet High, Masters in Chemistry**, Biomerieux: Advisor/Consultant|Biomerieux: Grant/Research Support **David Brealey, MBBSPhD**, Biomerieux: Honoraria **David Livermore, BSc PhD**, bioMerieux: Honoraria|bioMerieux: Provision of machines/test kits for INHALE.

